# Factors Affecting Acceptance of Hospital Information Systems in Public Hospitals of Zahedan University of Medical Sciences: A Cross-Sectional Study

**DOI:** 10.25122/jml-2019-0064

**Published:** 2019

**Authors:** Jahanpour Alipour, Yousef Mehdipour, Afsaneh Karimi

**Affiliations:** 1. Department of Health Information Technology, Paramedical School, Zahedan University of Medical Sciences, Zahedan, Iran; 2. Health Promotion Research Center, Zahedan University of Medical Sciences, Zahedan, Iran

**Keywords:** Affecting factor, Hospital information system, public hospital, HIS acceptance, Hospital information system adoption, usability

## Abstract

A hospital information system is used to support a wide range of operations and activities in the hospital. This study was conducted to determine the factors affecting hospital information system acceptance by users.

A cross-sectional, descriptive, analytic study was performed in 2018. The study population included 550 users of the system. The data were collected using a questionnaire and analyzed using the SPSS software.

A significant moderate positive correlation was found between hospital information system acceptance and perceived usefulness (r = 0.54, P < 0.01), perceived ease of use (r = 0.41, P < 0.01), human factors (r = 0.46, P < 0.01) and technological factors (r = 0.54, P < 0.01). A significant weak positive correlation was detected between the acceptance of the hospital information system and organizational factors (r = 0.35, P < 0.01).

Perceived usefulness of the system, social influence, system quality, perceived ease of use of the system, and top managers’ supports had the most substantial influence on the users’ intention to accept a hospital information system. User education, preparation of guidelines suited to the user specialty or department, incorporating users’ work needs into the capabilities of the hospital information system, and improving the system to an ideal level are important considerations.

## Introduction

The ultimate goal of health information systems is to encourage efficient and effective decisions through improving data quality and subsequently enhance the health care services [1]. Effective health service systems require continuous improvement in the health information system domain [2] because healthcare institutions, including hospitals, need to use a computerized information system to manage a large volume of data [3]. A hospital information system (HIS) is a computerized system used to manage the administrative, financial, and clinical activities of the hospitals [4]. Implementation and application of these systems lead to improved clinical processes and healthcare quality, decrease healthcare costs, and increase the healthcare providers’ and patients’ satisfaction [5].

The emergence of hospital information systems in Iran dates back to about two decades ago [6]. Now, all of the hospitals in Iran use HIS for data management [7]. Hospital information systems severely rely on data and information; hence, healthcare institutions have to know different health information system domains to understand their capabilities [8]. Handayani et al. found that organizational and human factors were the most critical factors in HIS acceptance [9]. Acceptance or rejection of a system by users is a determinant of the system’s success or failure [10-12]. Successful implementation of these systems depends on user satisfaction [13], the need for system perception, trust and sense of ownership of the users to the system, and participation of users in system development [10]. The inefficiency of the hospital information system in meeting the users’ needs results in the rejection of these systems; moreover, the users will also consider the system as an obstacle to their activities.

Despite the implementation and benefits of the HIS, health care professionals, as HIS users, do not use these systems entirely. Because of the critical role of these users in the success of information systems, there are concerns about the acceptance of these systems by the users [14]. Few studies have assessed health information technology acceptance in developing countries [15]. Many technology acceptance models have been developed and applied to assess and predict new information technologies, which are mainly general [14]. Thus, the use of a valid model for the evaluation of information system acceptance by its users is critical [11]. The technology acceptance model (TAM) is a valid theory that is widely used to model how users come to accept and use technology [11, 16-18]. Initially, TAM, as a general model, was developed by Davis for the investigation of new information technology acceptance from the perspectives of groups or organizations. However, this model has been used in many studies to determine the factors affecting the acceptance of health information technology and the relationship between factors [19].

Venkatesh and Davis introduced two factors of perceived usefulness and perceived ease of use as a pillar of TAM [20]. Handayani et al. used TAM to develop a new model for HIS evaluation that, in addition to the TAM factors, included technological, human, and organizational factors for the assessment of the external variables affecting HIS acceptance [19]. More than a decade after implementing hospital information systems in public hospitals affiliated to Zahedan University of Medical Sciences, it is necessary to evaluate these systems comprehensively to achieve the goal of applying these systems. Thus, this research was conducted to determine the factors affecting HIS acceptance by users in public hospitals of Zahedan University of Medical Sciences (ZAUMS).

## Material and Methods

This applied study was conducted in 2018, using a descriptive-analytical and cross-section method. The research population of this study consisted of the users of public hospitals affiliated with ZAUMS, including physicians (217) as well as nursing staff (1003), medical records or health information technology (49), laboratory (98), radiology (49), and pharmacy (19) staff working in Ali-Ibne-Abi Talib, Khatam-al-Anbia, Alzahra, Baharan, and Buali Hospitals. Sampling was done only for the nursing staff to select 276 samples using the Cochran formula. Because of population limitations in other groups, sampling was not done, and the whole population was selected as a sample.

A two-section questionnaire designed by Handayani et al. for the evaluation of hospital information systems in developing countries was used in this study [19]. The first section addressed the participants’ demographic data, including age, sex, job level, work experience, HIS experience, and education. The second part of the questionnaire contained 44 questions for the evaluation of HIS acceptance by users based on six dimensions. The dimensions were perceived usefulness (n=4), perceived ease of use (n=4), human factors (including four variables of compatibility (n=3), information security (n=4), self-efficacy (n=3), and social influence (n=3)), technological factors (including 2 variables of information quality (n=4) and system quality (n=4)), organizational factors (including 3 variables of top management support (n=4), participation of end-users in the HIS implementation process (n=4), and facilitating conditions (n=3)), and HIS acceptance (n=4). For each question, a five-point Likert scale (from 1 - very low to 5 - very high) was used to rate each sub-factor. The questionnaires were then validated by a panel of four health information management experts. The reliability of the questionnaire was examined using the internal consistency coefficient (Cronbach’s alpha= 0.88).

Descriptive (mean ± standard deviation) and analytic (Spearman and Pearson correlations) statistics were applied to analyze the data using the Statistical Package for Social Sciences (SPSS) software. The mean score of the dimensions was used to determine the desirability level of HIS acceptance by users. A mean score of ≥3.75, 3-3.75, 1.5-3, and <1.5 out of 5 was considered as desirable, relatively desirable, relative failure, and undesirable HIS acceptance, respectively.

## Results

Of the 550 participants who completed the questionnaires, 352 (64%) were female. Most of the users (65.7%) had bachelor’s degrees. The nursing staff comprised most of the respondents in the user population (46.4%), and the mean age of the participants was 33.57 ± 8.41 years. Most of the users had 1-6 years of experience (54.9 %), and more than half of them (52.7%) had 1-3 years of experience in work with HIS (Table 1).

**Table 1:** Respondent demographics.

Category		Total number	%^a^
**Sex**	Male	197	36
	Female	352	64
**Age**	21-27	164	30
	28-34	143	26
	35-41	163	30
	42-48	36	7
	49-56	44	8
**Job level**	Physician	121	22
	Nursing	245	45
	Laboratory	77	14
	Radiology	43	8
	Pharmacy	15	3
	Medical record	49	9
**Work experience**	1-6	302	55
	7-12	142	26
	13-18	40	7
	19-24	28	5
	25-30	38	7
**HIS experience**	1-3	275	50
	4-6	235	43
	7-10	40	7
**Education**	Diploma	5	1
	Associate’s Degree	11	5
	Bachelor’s	345	63
	Master’s	51	9
	PhD	97	5
	Professional doctor	26	18

a The percent of items were rounded

Table 2 shows that from the users’ perspective, the highest and lowest mean score of HIS determinants was related to perceived usefulness and organizational factors, respectively.

**Table 2:** The mean score and correlations of evaluated dimensions in the given hospital information system from the viewpoints of HIS users.

Variable		Mean±S.D	1	2	3	4	5	6
**1**	**PU**	3.86 ± 0.67	1.00**					
**2**	**PEOU**	3.73 ± 0.65	.64**	1.00**				
**3**	**Human**	3.44 ± 0.62	.60**	.55**	1.00**			
**4**	**Technology**	3.69 ± 0.67	.62**	.51**	.73**	1.00**		
**5**	**Organizational**	3.25 ± 0.69	.48**	.36**	.76**	.65**	1.00**	
**6**	**HIS acceptance**	3.85 ± 0.76	.54**	.41**	.46**	.54**	.35**	1.00**

PU: perceived usefulness, PEOU: perceived ease of use, **p < .01

According to Table 3, there is a significant, robust and positive correlation between perceived usefulness and perceived ease of use, perceived usefulness and information quality, self-efficacy and information security, self-efficacy and social influence, system quality and information quality, top management support and system quality, and facilitating conditions and compatibility of the system with users’ job requirements.

**Table 3:** The mean score and correlations of evaluated factors in the given hospital information system from the viewpoints of HIS users.

Variables		Mean±S.D	1	2	3	4	5	6	7	8	9	10	11	12
**1**	**PU**	3.86 ± 0.68	1.00											
**2**	**PEOU**	3.73 ± 0.65	.64	1.00										
**3**	**Comp**	3.43 ± 0.82	.38	.42	1.00									
**4**	**IS**	3.50 ± 0.76	.49	.45	.40	1.00								
**5**	**SE**	3.38 ± 0.73	.49	.46	.46	.60	1.00							
**6**	**SI**	3.35 ± 0.83	.54	.41	.38	.46	.65	1.00						
**7**	**IQ**	3.67 ± 0.75	.62	.54	.46	.56	.59	.58	1.00					
**8**	**SQ**	3.71 ± 0.73	.50	.38	.43	.48	.51	.56	.64	1.00				
**9**	**TMS**	3.56 ± 0.84	.50	.35	.53	.59	.42	.51	.53	.62	1.00			
**10**	**POEU**	2.76 ± 0.84	.28	.13	.38	.44	.39	.33	.39	.45	.50	1.00		
**11**	**F**C	3.43 ± 0.80	.38	.41	.99	.39	.45	.38	.45	.43	.52	.38	1.00	
**12**	**HIS-A**	3.85 ± 0.76	.54	.41	.28	.33	.47	.52	.47	.51	.34	.22	.28	1.00

PU: perceived usefulness, PEOU: perceived ease of use, Comp: compatibility, IS: Information security, SE: self-efficacy, SI: social influence, IQ: information quality, SQ: system quality, TMS: top management support, POEU: Participation of End-users in the HIS Implementation Process, FC: Facilitating conditions, HIS-A: HIS acceptance, **p < .01

## Discussion

### Perceived usefulness (PU)

“Perceived usefulness” refers to the user’s subjective beliefs of the effectiveness of a hospital information system to enhance his/her job performance in a healthcare facility [21]. In this study, the mean PU score was 3.86 ± 0.68, indicating the desirability of the system’s usefulness from the users’ perspective. Hence, users believed that HIS could enhance their job productivity, performance, and effectiveness. However, the promotion of PEOU, technological factors, human factors, HIS acceptance, and organizational factors can enhance the system’s usefulness to an ideal level.

Tabibi et al. [22], Farzandipour et al. [23], and Kamaludin et al. [24] reported a mean score of 3.71 ± 0.68, 3.45, and 2.81 ± 0.87 for PU, respectively. The findings of this study are consistent with the results of [22], [23], [25], but inconsistent with the results of [24] and [26]. This contradiction could be due to differences in the study population. The results of the current study indicated that system usefulness was mostly influenced by PEOU (r = 0.64, P < 0.01), technology (r = 0.62, P < 0.01), human factors (r = 0.60, P < 0.01), and HIS acceptance and less influenced by organizational factors (r = 0.48, P < 0.01). These findings are consistent with the results of previous studies [21, 27-29].

### Perceived Ease of Use (PEOU)

Perceived ease of use is defined as “the degree to which a person believes that using technology would be free from effort” [30]. In the context of this study, PEOU referred to the extent to which users believed that their continuous use of HIS was with ease. In this study, the mean score of PEOU was 3.73 ± 0.65, indicating the relative desirability of the system’s ease of use from the users’ point of view. The data also showed that PEOU was mostly affected by human (r = 0.55, P < 0.01) and technological factors (r = 0.51, P < 0.01) and was less influenced by HIS acceptance (r = 0.41, P < 0.01) and organizational factors (r = 0.36, P < 0.01). Moreover, the study results showed that the variable of information quality (r = 0.54, P < 0.01) in technological factors had a significant impact on users’ perceived HIS ease of use.

PEOU, information quality, users’ computer self-efficacy, information security, compatibility of systems with users’ job requirements, facilitating conditions, users’ social influence, system quality and participating of users in the development of HIS had the most significant influence on users’ perceived HIS ease of use in the mentioned order. Hence, improving the interpretability, comprehensiveness, and accuracy of the information in HIS, as well as improving HIS compatibility with the users’ job requirements, information security, and self-efficacy and social influence of the users, may enhance the perception of the system’s ease of use.

Farzandipour et al. [23], Tabibi et al. [22], and Kamaludin et al. [24] reported a mean score of 3.47, 4.03 ± 0.55, and 3.33 ± 0.77 for PEOU, respectively. The findings of this study are in line with the results of [23] and [24] but inconsistent with the results of [22]. These contradictions may be explained by the knowledge of users as well as the quality of the system and the information contained in the systems used in these studies.

### Technological factors

Previous studies [9, 19, 27] used two measures for the evaluation of technological factors, including system quality and information quality. Technological factors included the variables of system quality and information quality, which were related to HIS capabilities [19]. In this study, the mean score of technological factors was 3.69 ± 0.67, indicating the relative desirability of the HIS in terms of technology from the users’ point of view. A significant, strong, positive correlation was found between technological factor and perceived usefulness (r = 0.62, P < 0.01), human factors (r = 0.73, P < 0.01), and organizational factors (r = 0.76, P < 0.01), and a moderate positive correlation was found between technological factors and the system’s ease of use (r = 0.53, P < 0.01), and HIS acceptance (r = 0.54, P < 0.01).

Jabraeily et al. [31], Aggelidis and Chatzoglou [32], and Mahdavian et al. [33] reported a mean score of 4.35 ± 0.71, 3.56 ± 0.72, and 3.48 ± 0.44 for technological factors, respectively. The results of this evaluation are in accord with [32] and [33] but inconsistent with [31]. This inconsistency can be due to differences in the measures used for the evaluation of technological factors.

System quality is defined as the degree to which the system or software has features such as being free of bugs, user interface consistency, ease of use, system responsiveness in interactions, system documentation and quality, and the ability to maintain programming codes [34]. In the context of this study, system quality referred to the extent to which the system or software had features such as covering all job functions of the users, system response speed, and system availability for service provision without time constraints. This results showed that system quality was mostly influenced by information quality (r = 0.64, P < 0.01) in the technological factors, top management support (r = 0.62, P < 0.01) in organizational factors, social influence (r = 0.56, P < 0.01) and self-efficacy (r = 0.51, P < 0.01) in human factors, and HIS acceptance (r = 0.51, P < 0.01).

The quality of the information implies that the system information has desirable characteristics such as relevance, understandability, accuracy, conciseness, completeness, currency, timeliness, and usability [34]. In the context of this study, information quality referred to understandability, comprehensiveness, accuracy, timeliness, relevance, legibility, and consistency of the patients’ information and reports produced by the system. This study showed that information quality was strongly influenced by system quality (r = 0.64, P < 0.01) in technological factors and perceived usefulness (r = 0.62, P < 0.01), and influenced moderately by self-efficacy (r = 0.59, P < 0.01) and social influence (r = 0.58, P < 0.01) in human factors, system’s ease of use (r = 0.54, P < 0.01), and top management support (r = 0.53, P < 0.01) in organizational factors.

### Human Dimension

Human capital plays a crucial role in every organization. Hence, the adaptation of specialists is essential for the success of a system [35]. Previous studies [9, 19] used four measures for the evaluation of human factors, including compatibility, information security, self-efficacy, and social influence. The same measures were used in this study.

The mean score of the human factors was 3.69 ± 0.67 in this study, indicating the systems were relatively satisfactory in terms of human factors from the users’ perspective. Ghaderi Nansa et al. [36] reported a mean score of 2.96 for the compatibility of the system with user expectations. Lee et al. [37] introduced human factors as an essential external determinant of HIS adoption. Vollmer et al. [38] established a mean score of 3.19 ± 0.90 and 2.95 ± 0.97 for social influence in the University Hospital in Erlangen and the IMH Medical Center, respectively. Aggelidis and Chatzoglou [16] reported a mean score of 4.0 ± 0.68 and 3.6 ± 0.66 for social influence and self-efficacy, respectively. Jabraeily et al. [31] reported a mean score of 4.22 ± 0.69 for human factors. The results of [36] and [38] are relatively consistent, while [16], [31] are inconsistent with the findings of the present study. These discrepancies can be explained by differences in the measures used for the evaluation of human factors in these studies.

A significant, strong, positive correlation was found between human factors and organizational (r = 0.76, P < 0.01) and technological factors (r = 0.73, P < 0.01). Moreover, a moderate positive correlation was found between human factors and the system ease of use (r = 0.55, P < 0.01), perceived usefulness (r = 0.60, P < 0.01), and HIS acceptance (r = 0.46, P < 0.01).

Self-efficacy refers to the extent to which the users of an information system feel able to complete the tasks of the job using the system [39]. In this study, self-efficacy referred to the ability of the users of the hospital information systems to complete the tasks using these systems. Social influence, information quality, system quality, perceived usefulness, HIS acceptance, perceived ease of use, compatibility, facilitating conditions, top management support, and participating of the end-user in the system implementation had the most significant influence on the users’ self-efficacy, respectively.

Compatibility is defined as the degree to which an innovation is perceived as being consistent with the existing values, needs, and experiences of potential adopters [39]. In this study, compatibility referred to the concordance of the hospital information system with user needs, functioning, and task priorities. A substantial significant positive correlation was found between compatibility and facilitating conditions (r = 0.99, P < 0.01), indicating that facilitating conditions such as user manuals with clear instructions on how to use HIS based on specialized units or personnel and adequate supporting resources (i.e., software, hardware, networks) have a substantial effect on the users’ tendency to adopt a HIS.

Information security is defined as processes designed to protect the information from unauthorized access and preserve the accuracy and completeness of information [40]. In this study, information security referred to features such as confidentiality, accessibility, compatibility, and prevention of unauthorized access to information or its modification. Self-efficacy (r = 0.60, P < 0.01), top management support (r = 0.59, P < 0.01), and information quality (r = 0.56, P < 0.01) had the greatest influence on information security, respectively. These results showed that the users’ skills and capabilities of using HIS, HIS output quality, and senior managers’ supports affected HIS adoption by users.

Social influence refers to the extent to which a user perceives that other important people believe he or she should use the new system [41, 42]. In this study, the social influence referred to the degree to which the beliefs of other important people for users (such as peers, friends, and others) affected the user’s adoption of the hospital information system. The results of our study specified that social influence was mostly affected by self-efficacy (r = 0.65, P < 0.01), information quality (r = 0.58, P < 0.01), system quality (r = 0.56, P < 0.01), perceived HIS usefulness (r = 0.54, P < 0.01), top management support (r = 0.51, P < 0.01), and HIS acceptance (r = 0.52, P < 0.01).

### Organizational Dimension

Regarding the adoption of new technologies, organizational factors are one of the critical factors that influence the successful adoption of health information systems [9, 19, 35, 37, 43-45]. In the present study, similar to studies by Handayani et al. [9, 19], three measures of facilitating conditions, top management support, and user involvement in HIS implementation were used for HIS evaluation in terms of organizational factors. The mean score of organizational factors was 3.25 ± 0.69, indicating the relative desirability. Likewise, our results showed that organization factors had a significant, strong positive correlation with the human (r = 0.76, P < 0.01) and technology (r = 0.65, P < 0.01) factors, a significant, moderate positive correlation with PU (r = 0.48, P < 0.01), and a significant, weak positive correlation with PEOU (r = 0.36, P < 0.01) and HIS acceptance (r = 0.35, P < 0.01).

Facilitating conditions are defined as “objective factors in the environment that observers agree to make an act easy to do, including the provision of computer support” [39]. In this context, facilitating conditions referred to user manuals containing clear instructions on how to use a HIS based on specialized units or personnel and adequate supporting resources such as software, hardware, and networks.

A very strong significant positive correlation was found between facilitating conditions and HIS compatibility (r = 0.99, P < 0.01). This finding showed that user access to dedicated guides and hardware, software, and network resources could have a significant impact on the HIS compatibility for users.

The perceived importance of health information technology by top managers and their support has a crucial role in information adoption in hospitals [44, 46-50]. Top management support refers to the degree to which executive managers perceive the nature and function of new technology and fully support its development [35]. In this framework, top management support also refers to the degree that top managers fully support HIS innovation and provide a suitable task environment for working with HIS. System quality, information security, system compatibility with user expectations, information quality, facilitating conditions, social influence, PU, the participation of users in the development of HIS, self-efficacy, PEOU, and HIS acceptance had a significant impact on the top management support, respectively.

User participation in HIS implementation is defined as “the active participation of HIS users in the communication, design, implementation, and training processes of HIS implementation” [19]. End-user involvement in the development or implementation of an information system can improve the success of the system through enhancing the user’s perception of the system content and objectives, feelings ownership of the system, and enhanced concordance between user information needs and system capabilities [10, 51]. A, moderate, significant, positive correlation was found between user participation in HIS implementation and top management support (r = 0.50, P < 0.01), system quality (r = 0.45, P < 0.01), and information security (r = 0.44, P < 0.01).

Farzandipour et al. [23], Jabraeily et al. [31], and Tavakoli et al. [52] reported a mean score of 2.42, 3.98 ± 0.72, and 59.80 ± 13.05 for organizational factors, respectively. Aggelidis and Chatzoglou [16] reported a mean score of 3.69 ± 0.68 for facilitating conditions. Vollmer et al. [38] reported a mean score of 3.82 ± 0.56 and 3.82 ± 0.63 for facilitating conditions in the University Hospital in Erlangen and the IMH Medical Center, respectively. Regarding organizational factors, our findings were almost similar to the results of most of the previous studies but inconsistent with [23]. This contradiction may be due to differences in the tools used for the evaluation of user participation in HIS implementation.

## Conclusion

Although the users’ intentions to accept HIS were at a desirable level, only perceived usefulness of the system was at a satisfactory level, while perceived ease of use, human factors, technological factors, and organizational factors were at a relatively desirable level from the users’ perspective. Perceived usefulness of the system, social influence in human factors, system quality in technological factors, perceived ease of use, and top managers’ support in organizational factors had the highest impact on users’ intention to accept a HIS. Simplifying the use of the system through educating users and providing comprehensive and special guidelines suited to the user’s specialty or department, incorporating users’ work needs into HIS capabilities, and involving users in the development, implementation, and education steps of the HIS software are essential to upgrade the system to an ideal level, increase user satisfaction, and enhance the system acceptance to an optimal level.

## Conflict of Interest

The authors confirm that there are no conflicts of interest.

## Funding

This study was supported by the Vice-Chancellor for Research and Technology of Zahedan University of Medical Sciences [Grant No. IR.ZAUMS.REC.1397.124].Guarantor: JA

## Ethical approval

The ethics committee of IR.ZAUMS.REC approved this study (REC.1397.124)

## Acknowledgment

The authors would like to thank the Vice-Chancellor for Research and Technology of Zahedan University of Medical Sciences for supporting the project. The authors also thank HIS users of the given hospital information systems who participated in this study for sharing their valuable experiences.
